# cGAS-STING Pathway Performance in the Vulnerable Atherosclerotic Plaque

**DOI:** 10.14336/AD.2022.0417

**Published:** 2022-12-01

**Authors:** Xueqi Wan, Jinfan Tian, Peng Hao, Kuo Zhou, Jing Zhang, Yuquan Zhou, Changjiang Ge, Xiantao Song

**Affiliations:** Department of Cardiology, Beijing Anzhen Hospital, Capital Medical University, Beijing, China

**Keywords:** atherosclerosis, plaque vulnerability, STIM1, STING, Ca^2+^

## Abstract

The important role of Ca^2+^ in pathogenic store-operated calcium entry (SOCE) is well-established. Among the proteins involved in the calcium signaling pathway, Stromal interacting molecule 1 (STIM1) is a critical endoplasmic reticulum transmembrane protein. STIM1 is activated by the depletion of calcium stores and then binds to another calcium protein, Orai1, to form a channel through which the extracellular Ca^2+^ can enter the cytoplasm to replenish the calcium store. Multiple studies have shown that increased STIM1 facilitates the aberrant proliferation and apoptosis of vascular smooth cells (VSMC) and macrophages which can promote the formation of rupture-prone plaque. Together with regulating the cytosolic Ca^2+^ concentration, STIM1 also activates STING through altered intracellular Ca^2+^ concentration, a critical pro-inflammatory molecule. The cGAS-STING pathway is linked with cellular proliferation and phenotypic conversion of VSMC and enhances the progression of atherosclerosis plaque. In summary, we conclude that STIM1/cGAS-STING is involved in the progression of AS and plaque vulnerability.

## 1. Introduction

Atherosclerosis (AS) is a major cardiovascular malignancy [1]. Although there are several treatment strategies for AS in the clinic, a high risk of cardiovascular complications such as myocardial infarction, myocardial ischemia, heart failure, and stroke are often reported in different subgroups of AS patients [2]. Therefore, it is critical to identify novel proteins and signaling pathways that can be targeted to alleviate this debilitating disease.

Unstable plaque rupture leads to severe complications such as thrombosis, which can be lethal, so the therapies that increase the stability are urgently needed for patients suffering from cardiovascular diseases (CVD). Previous studies found that the vulnerability of the plaque is dependent on the components instead of the size [3]. As AS progresses, many factors cause endothelial damage, such as dysregulated proliferation and apoptosis of vascular smooth muscle cells (VSMCs), inflammatory cascade, infiltration of macrophages into the endothelial interval, etc. [4, 5]. The uncontrolled proliferation of vascular smooth muscle cells is not only a crucial factor in the pathogenesis of atherogenesis but also the main initiating factor of vascular intima thickening. Aberrant proliferation and apoptosis of VSMC not only make it difficult to remove apoptotic foam cells but also lead to the reduction in the secretion of collagen and thinner fibrous cap [6-8], aggravating the vulnerability of the plaque. The inflammatory response then recruits macrophages to the subintima, and the macrophages accumulating become foam cells by engulfing ox-LDL and other lipids with the assistance of scavenger receptors [9-11]. These macrophages, in turn, continue to release inflammatory factors, exacerbating the inflammatory response [12].

Intracellular calcium ion (Ca^2+^) is a ubiquitous mediator of cell proliferation and apoptosis, which are closely correlated to vascular remodeling [13, 14]. The increase in free Ca^2+^ concentration can direct cell proliferation, but excessive increase or even depletion of endoplasmic reticulum Ca^2+^ can lead to cell apoptosis. Intracellular Ca^2+^ homeostasis is maintained by Na^+^/Ca^2+^ exchanger, Ca^2+^ pumps and Ca^2+^ channels; the opening and activation of such pumps are associated with atherosclerotic vascular diseases [3]. During the progression of atherosclerotic plaque formation, aberrant cellular Ca^2+^ channel proteins inevitably lead to abnormal intracellular Ca^2+^ levels. This promotes cell proliferation and apoptosis, which result in vascular remodeling, and plaque damage, accelerating the process of acute coronary events.

**Figure 1.**
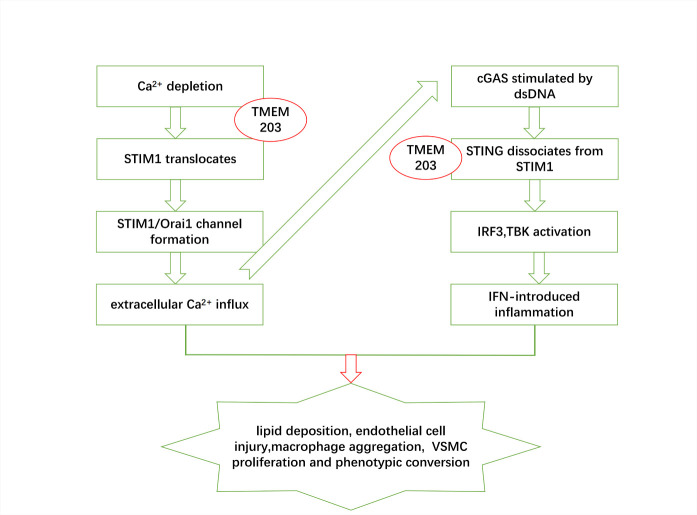
The involvement of cGAS-STING, as well as STIM1, in various of steps toward the developments of AS. During the progression of atherosclerosis, overexpression of TMEM203 promotes stored Ca^2+^ depletion, which in turn promotes STIM1 dissociation from STING and its translocation to the cell membrane to bind to Orai1. These lead to elevated intracellular calcium ion concentration and promotion of the atherosclerotic process. On the other hand, elevated intracellular calcium ions lead to direct activation of cGAS-STING, or competitive binding of TMEM203 to STING promotes its dissociation of STIM1 from STING. Activated STING promotes IFN-mediated inflammatory responses. Thus, STIM1 and STING promote lipid deposition, endothelial cell injury, macrophage aggregation, smooth muscle cell proliferation, and phenotypic transformation.

The Ca^2+^ influx via store-operated calcium channel (SOCC) plays an important role in the Ca^2+^ homeostasis of the circulatory system [13]. Stromal interacting molecule 1 (STIM1), an important component of SOCC, can bind to the Stimulator of interferon genes (STING). STIM1 inhibits the activation of STING by retaining STING in the endoplasmic reticulum and blocking the translocation of STING to the Golgi body [15]. Currently, an increasing number of studies are focused on investigating the relationship between AS and activation of cyclic GMP-AMP (cGAMP) synthase (cGAS)-STING based on the role of cGAS-STING pathway performance in initiating inflammatory responses and the presence of the inflammatory milieu in AS. To our surprise, a newly published article by Bi Xi et al. [6] showed that overexpression of cGAS-STING inhibited the proliferation of VSMC but promoted their premature senescence and phenotypic switching in chronic kidney disease (CKD)/ApoE-/- mice. The result may provide new insight for our idea. Therefore, in this review, we postulate that STIM1 promotes the progression of AS and plaque vulnerability, partly by regulating the initiation of cGAS-STING signaling (Fig. 1).

## 2.STIM1/cGAS-STING signal pathway

### 2.1 Structure of STIM1, cGAS, and STING molecule

Stromal interaction molecule 1 (STIM1), residing in the endoplasmic reticulum membrane, is an important member of the molecular machinery of SOCE and acts as a Ca^2+^ sensor. STIM1 has an N-terminal Ca^2+^ binding domain facing the endoplasmic reticulum cavity with low affinity [16]. Upon stimulation, G-protein-coupled receptors are activated, which activates the phospholipase C (PLC) and the formation of inositol 1,4,5-trisphosphate (IP_3_). IP_3_ then binds with the IP_3_ receptor (IP_3_R), opening the Ca^2+^ release channel in the ER and facilitating release of Ca^2+^. On sensing the depletion of ER Ca^2+^, STIM1 translocates into the membrane junction between ER and plasma membrane, activating Orai1 channel or transient receptor channel (TRPC), which results in the influx of Ca^2+^ [17-20].

cGAS is a cytosolic DNA sensor that gets activated upon binding with cytoplasmic DNA. STING, located in the endoplasmic reticulum membrane, contains four transmembranes (TMs) segments at its N-terminal. TM segments of STING N-terminal domain (NTD) are involved in ER localization and transportation. The cytoplasmic domain (CTD) of STING includes dimer domain (DD), cyclic dinucleotides (CDN) binding region with high affinity, and C-terminal tail (CTT), which interacts with TBK1 and IRF3 [21-23]. STING also contains Ca^2+^ binding sites in certain sequences [22].

### 2.2 The activation of STING signaling

#### 2.2.1 The interaction of between Ca^2+^ and STING

The STING dimers contain two Ca^2+^ binding sites, and a certain level of cytosolic Ca^2+^ seems to be necessary for its function [22, 24]. An example of DNA-dependent STING activities in macrophages is the reduction of IRF3 and NF-kB activity by three Ca^2+^ blocking reagents-BAPTA-AM, CsA, or CGP37157. The inhibition of NF-kB and IRF3 cytokine secretion can also be reversed by agents that increase the cytosolic Ca^2+^ such as valinomycin and thapsigargin [25]. TypeIIFN expression, induced by STING activation, is sensitive to intracellular Ca^2+^ levels [26]. Moreover, Ca^2+^ signaling is shown to be a prerequisite for activation of STING and IRF3 during HCMV (human cytomegalovirus) invasion [27]. The potential mechanism by which cytosolic Ca^2+^ promotes STING activity is as follows: calcium influx initiates Calcium/calmodulin dependent protein kinase II (CAMKII), which leads to the stimulation of phosphorylates 5’ AMP-activated protein kinase (AMPK) [28]. AMPK decreases ULK1, which lowers STING levels [29]. Paradoxically, cytosolic Ca^2+^ elevation induced by ionomycin can also inhibit STING activation [30]. Therefore, an optimal Ca^2+^ concentration is a key factor in regulating the basal and activation states of STING.

#### 2.2.2 cGAS-STING signaling

Upon stimulation by dsDNA generated by mitochondrial damage, genomic instability, or exogenous microbe, cGAS utilizes ATP and GTP to synthesize the only known metazoan cyclic-dinucleotide, cGAMP. Subsequently, cGAMP acts as a second messenger diffusing and binding to the ER membrane-bound adaptor protein STING, thus priming its translocation into the ER-Golgi intermediate compartment (ERGIC) to recruit TANK-binding kinase 1 (TBK1) and interferon regulatory factor 3 (IRF3). IRF3, upon phosphorylation by TBK1, homo-dimerizes and translocates into the nucleus to induce transcription of type I IFNs, followed by the inflammatory cascade reaction, which exacerbates pathological cell proliferation and migration [23, 31, 32].

#### 2.2.3 STIM1, a regulator for cGAS-STING activation via Ca^2+^

**Figure 2.**
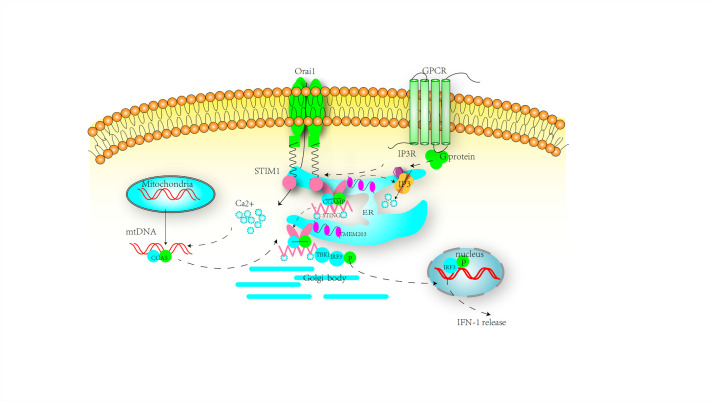
The activation process of the STING pathway. Under resting state, STIM1 binds to STING, suppressing its exit from ER, a critical step in STING’s initiation of downstream type I interferon signaling. TMEM203 binds to IP_3_R and STIM1 to maintain cellular calcium homeostasis. Under pathological conditions, overexpression of TMEM203 leads to a depletion of endoplasmic reticulum (ER) calcium stores. The activation of TMEM203 directly promotes the dissociation of STING from STIM1, followed by the activation of the STING cascade. On the other hand, alteration of cellular calcium concentration indirectly activates the STING cascade. Firstly, the calcium store depletion induced by overexpression of TMEM203 facilitates the translocation of STIM1 and retains more STIM1 protein for binding to Orai1. Secondly, upon stimulation by mitochondrial damage generated dsDNA, and induced by elevated intracellular calcium concentration, cGAS utilizes ATP and GTP to synthesize cyclic GMP-AMP (cGAMP). Subsequently, cGAMP acts as a second messenger, diffusing and binding to STING, thus priming its translocation into the ER-Golgi intermediate compartment (ERGIC) to recruit TANK-binding kinase 1 (TBK1) and interferon regulatory factor 3 (IRF3). IRF3, upon phosphorylation by TBK1, homo-dimerizes and translocates into the nucleus to induce transcription of type I IFNs, followed by the activation of the inflammatory cascade.

STIM1, acts as an ER retention factor of STING in the ER membrane. Under basal state, STIM1 binds with STING to retain it in the endothelial reticulum (ER) membrane suppressing its exit from ER, a critical step for STING’s initiation of downstream type I interferon signaling (Fig. 2) [21, 33]. Prabakaran Thaneas et al. [34] found that ISD017 inhibited STING-induced IFN-1 in mice spleen. Moreover, they identified that in STIM1 deficient macrophages, the amount of precipitation of STING with this peptide apparently reduced, displayed by the crosstalk technology, which confirmed that the suppressive effect of ISD017 is dependent on the STIM1 target. STIM1 is an essential protein in SOCE and significantly changes the Ca^2+^ homeostasis. Cytosolic Ca^2+^ elevation mediated by ER calcium store depletion results in mitostress and mtDNA release and cGAS-STING mediated innate responses [35].

To further elucidate the mechanism of STIM1 and cGAS-STING activation, a novel protein TMEM203 was discovered. In previous functional screens, TMEM203 was identified as a proinflammatory gene in macrophages, acting as a binding partner and regulator of STING-mediated inflammatory signaling [36]. Further exploration demonstrated that TMEM203, interacting with the pleiotropic inositol phosphate signaling pathway protein IP_3_R and other components of SOCE, is a modulator of cellular calcium Ca^2+^ homeostasis, similar to STIM1. Under pathological conditions, TMEM203 actives STING pathway directly or indirectly. Upon stimulation, it forms a functional and ligand-dependent complex with STING. Over-expressed TMEM203 directly accelerates the dissociation of STING from ER competing with STIM1. On the contrary, the downregulation of TMEM203 negated the release of IFN-1 and the expression of downstream proteins such as IRF3 [23]. Further, overexpression of TMEM203 independently activated the STING pathway since activated TMEM203 depleted ER calcium stores [23, 37]. The calcium store depletion facilitates the dissociation of STIM1 from STING and translocation of STIM1 to the cell membrane to bind to Orai1, resulting in elevated intracellular Ca^2+^. Induced by mitochondrial damage generated dsDNA and elevated intracellular calcium concentration, cGAS utilizes ATP and GTP to synthesize cyclic GMP-AMP (cGAMP). Subsequently, cGAMP acts as a second messenger, diffusing and binding to the STING, thus priming the translocation into the ER-Golgi intermediate compartment (ERGIC) to recruit TANK-binding kinase 1 (TBK1) and interferon regulatory factor 3 (IRF3) (Fig. 2). In summary, the mechanism by which STIM1 regulates the STING pathway may be closely related to the activity of TMEM203 and cytosolic Ca^2+^ balance.

## 3.STIM1 for AS plaque

Macrophages, smooth muscle cells, platelets, and endothelial cells are critical players in the atherosclerotic process. During the activation of cGAS-STING, dissociated STIM1 from STING promotes atherogenesis and the development of atherosclerosis. Figure 1 shows STIM1 contributes to endothelial inflammation, lipid accumulation, and migration of vascular smooth muscle cells via extracellular Ca^2+^ influx or cGAS-STING activation.

### 3.1 Macrophage

Oxidized low-density lipoprotein (ox-LDL) can enhance the adhesion of monocytes and induce the transformation of monocytes into macrophages [38]. The ox-LDL in inflammatory sites is more likely to be phagocytosed and is critical for forming foam cells. As a highly inflammatory and cytotoxic substance, it can recruit macrophages, platelets, and other types of cells to release a variety of growth factors and interleukins, resulting in vascular smooth muscle cell proliferation, platelet aggregation, and thrombosis. Multiple studies have shown the importance of macrophages in AS progression and plaque vulnerability [9, 34, 38, 39]. Both *in vitro* and *in vivo* studies have found that STIM1 knockout reduced the increase of Ca^2+^ concentration in ox-LDL-induced macrophages, while genetic invalidation or pharmacological inhibition of STIM1/Orai1-mediated Ca^2+^ pathway attenuated the progression of atherosclerosis [9].

### 3.2 VSMC

Since several studies found STIM1 to be involved in AS, research has been focused on agents targeting STIM1. Ma et al. [40] observed that STIM1 facilitated proliferation, migration, and invasion of human-derived VSMC exposed to ox-LDL, which were attenuated by LncRNA MIAT inhibition or miR-641 upregulation in a STIM1-dependent manner. Another study demonstrated a novel crico-RNA targets STIM1 by binding with the micro-RNA. Increased proliferation and migration of ox-LDL challenged VSMC triggered by *Hsa_circ_0029589 knockdown or* miR-214-3p overexpression can be attenuated by downregulation of STIM1 [1]. Johnson MT et al. [41] showed that LCCB (L-type Ca^2+^ channel (Cav1.2) blockers) accentuates the STIM1 dependent remodeling of VSMC. Compared with control groups, the proliferation and migration triggered by amlodipine are significantly improved in VSMC treated with STIM1 genetic invalidation. AngII, a crucial hormone factor in the circulating system, induces aberrant pathological events of VSMC and severe vascular injury by activating inflammation. Egr-1 (Early growth response protein-1) was shown to participate in most of the anomalies triggered by angII. Interestingly, the gene silencing of STIM1 negated the effect of Egr-1 mediated by angII and Ca^2+^ elevation. Collectively, STIM1 promotes the unusual phenotype of VSMC in AS [17].

### 3.3 Platelet (PLT)

Matrix metalloproteinase-9 (MMP-9), an enzyme produced by infiltrating macrophages and VSMC, has been shown to play an important character in plaque development and pathogenesis of atherosclerosis [42, 43]. During the progression of AS, PLT can be activated by thrombin and release mediators such as platelet-derived growth factor (PDGF)-BB. These mediators are capable of stimulating VSMCs to migrate from the vessel media to the intima and secrete MMP-9 [44-46]. Xia W, et al. [47] discovered the high expression of SOCE associated components, such as STIM1, was correlated with peripheral vascular diseases in diabetes patients, which suggests that STIM1 may play an important role in PLT signaling pathways.

### 3.4 Endothelium

Previous evidence has shown the involvement of the endothelium damage in AS. Abnormal proliferation and apoptosis of endothelial cells are associated with endothelial perturbation, damage, and repair, contributing to the premature development of atherosclerosis and vascular complications in diabetes. The damage caused by high glucose exposure was found to be intertwined with Ca^2+^ oscillation. Bai S et al. [48] discovered that the human coronary artery endothelial cell migration and dysfunctional endothelial barrier function can be restored by SOCE inhibitor BTP-2 [48], which selectively inhibits STIM1. The endothelial progenitor cells (EPCs) have been shown to perform an important role in endothelial repair preventing the development of AS. In another study aiming to figure out the interplay between AS and SOCE, decreased Orai1, STIM1, and TRPC1 in atherosclerotic-EPCs were found compared with the control group. Further, the gene silencing of STIM1 or treatment with inhibitors 2-aminoethoxydiphenylborane (2-APB) and 1-(5-chloronaphthalene-1-sulfonyl)-1Hhexahydro-1,4-diazepine hydrochloride (ML-9), resulted in the downregulation of VEGF-induced eNOS expression and phosphorylation, a key protein involved in the biological functions of EPCs, SOCE amplitude as well as proliferative and migratory activities in EPCs [49]. Hence, the two experiments showed the different functions of STIM1 in endothelial function maintenance. These articles show that STIM1 may contribute to AS advancement by targeting the endothelium.

## 4.cGAS-STING pathway for AS plaque

cGAS-STING is a crucial player in inducing IFN-1 release for initiation of inflammatory responses in macrophage or other cell types. Therefore, several studies have shifted attention to investigating the function of cGAS-STING in AS plaque formation. Transactive response DNA-binding protein-43 (TDB43) enhances the inflammation and lipid uptake in macrophage challenged. In contrast, the siRNA-mediated cGAS silencing ameliorated the effect of TDB43 in macrophages [50]. A previous study exhibited the alleviation of the atherosclerotic lesion by genetically deleting STING and reducing macrophage accumulation in ApoE KO mice on a high-fat diet (HFD) [51]. Numerous studies have demonstrated that the promotion of AMPK and the suppression of the STAT1-STING pathway mediates anti-inflammatory responses [52]. Cai D et al. [12] showed that Balasubramid derivative 3C attenuated the activation of STAT1-STING by activating AMPK, which reduced the total cholesterol (TC) level in the serum and the necrosis core of plaque of ApoE-/- mice on a HFD. The *in vitro* blockade of STAT1-STING reduced the lipid accumulation in macrophages challenged with ox-LDL. To investigate whether the cCAS-STING activation is deleterious for AS plaque progression, Pham PT et al. [53] proved that cGAMP and STING profiles were increased in HFD-fed ApoE-/- mice. Reduction in plaque lesions and lipid accumulation were seen in mice treated with a specific antagonist of STING, C-167. *In vitro* studies showed that the STING agonist-treated macrophages showed a conspicuous increase in inflammatory factors including TNF-α, CCL-2, IFN-β, and downstream molecules TBK1 and NF-KB. Similarly, a rise in CGAMP and STING expression was observed in plaque specimens from patients undergoing carotid endarterectomy; the macrophages isolated from these patients also released more inflammatory factors. A downstream molecule-IRF3 induced by STING was also examined in the AS plaque. A higher IRF3 profile was seen in macrophages from atheromatous plaques in patients with coronary heart disease (CHD) and ApoE-/- mice. Meanwhile, the ablation of IRF3 in vivo attenuated the plaque lesion and infiltrated macrophage enriching in lipid. Expectedly, several canonical inflammatory markers such as IL-6, IL-1β, TNF-α showed higher expression in plaque [54]. These data imply that the increased activity of the cCAS-STING pathway promotes the AS plaque formation by increasing ox-LDL uptake into macrophages and up-regulation the inflammation pathway.

Growing evidence has confirmed the critical role of VSMC in both the initiation and progression of a rupture-prone plaque [7, 8]. The less collagen VSMC secretes, the thinner the fibrous cap is, which may lead to an unstable plaque. A newly published study in CKD mice demonstrated that the cGAS-STING activation, orchestrated by mitochondrial damage, exacerbated the phenotypic conversion and premature senescence of VSMC, producing less collagen, resulting in thinner fibrous cap and atherosclerotic plaque vulnerability. By contrast, knockout or knockdown of cGAS and STING dramatically decreased CKD-induced IFN-I response, premature senescence, and phenotypic switching [6].Similarly, telomere damage mediated senescence and inflammation in VSMC. Immunocytochemistry showed that the telomere damage leads to VSMC anomalies through the cGAS-STING-TBK1 pathway [55]. Furthermore, the deficiency of STING-activated IRF3 in ApoE -/- mice increased the smooth muscle cells content and fibrous collagen deposition, promoting the plaque stability identified by Oil Red O staining and scoring versus those of controls [54]. Another study exhibited that high fat upregulated the expression of cGAS and STING proteins. The activation of the cGAS-STING pathway accelerated inflammation and heart disease in HFD-fed ApoE-/- mice [56]. On the other hand, inhibiting STING down-regulated TNF-α, IFNα, and IFNβ, and decreased cardiac inflammation [57].

Recently, several studies found a specialized immune response to be involved in the progression of atheroma plaque formation [58, 59]. Interestingly, the cGAS-STING pathway has been elucidated to modulate the cellular immune response to viral and bacterial infections, and STING mutation or excessive STING activation is closely associated with various autoimmune diseases such as STING-associated vasculopathy with onset in infancy (SAVI) and systemic Lupus Erythematosus (SLE) [34, 60]. These observations indirectly support the connection between the STING pathway and AS plaque rupture.

## 5.Possible regulating mechanism of STIM1/cGAS-STING for AS plaque

Despite mounting evidence of activation of either STIM1 or cGAS-STING pathway in undermining the AS plaque stability, little is known how STIM1/cGAS-STING and downstream response are modulated. Even the mutual activation of STING and STIM1 for AS progression may be contradictory to the preliminary ideas that the STIM1 regulates the STING negatively. Based on the analysis in this review, we hypothesize that during the progression of AS, the overexpression of TMEM203 stimulates the activation of STING and downstream cascade such as IFN-1 secretion via directly promoting STING dissociation from STIM1 [23, 37]. Moreover, alteration of intracellular Ca^2+^ concentration indirectly contributes to the activation of STING cascade via the two mechanisms: Firstly, the calcium store depletion induced by over-expressed TMEM203 facilitates the translocation of STIM1 and retains more STIM1 protein for binding to Orai1 [35]. Secondly, the elevated intracellular Ca^2+^ promotes the spontaneous mtDNA production followed by the activation of the cGAS-STING cascade. Throughout the process, both STING (dissociated from STIMI1) and STIM1 promote atherosclerosis development. Therefore, it is plausible that cGAS-SING pathway is activated through over-expressioin of TMEM203 and altered intracellular Ca^2+^ concentration. Subsequently, aberrant cell apoptosis, proliferation, and inflammatory responses are set off to strengthen the instability of plaque.

## 6.Summary and perspective

Atherosclerosis is a malignancy whose complications caused by the atherosclerotic plaque rupture are lethal. Although various AS treatments (drug and intervention therapeutics) partly improve the prognosis of the disease, the morbidity and mortality in AS patients remain high. The Ca^2+^ homeostasis is regulated by Ca^2+^ channels. STIM1 acts as a channel activator and orchestrates the development of AS and exacerbates the plaque instability. Under normal conditions, STIM1 affects STING inversely. However, during the progression of AS microenvironment, STIM1 dissociates from STING, followed by the activation of the cGAS-STING pathway. Thereby, it is reasonable to conclude that STIM1 promotes proliferation, apoptosis, and inflammation via cGAS-STING, thus promoting AS plaque vulnerability. However, the underlying specific mechanisms are still not clearly understood. Hence, in-depth exploration is required to delineate the role of the STIM1/cGAS-STING signaling pathway in atherosclerotic plaque extension and vulnerability. The exact mechanism of its involvement in atheroma extension and vulnerable plaque formation needs to be clarified in future studies. This will shed light on the pathology and development of AS and vulnerable plaques. Exploration of novel AS drugs targeting this pathway is needed, which may help in avoiding acute coronary adverse events in AS patients.
